# *Porphyromonas gingivalis* as a *keystone* pathogen in systemic diseases

**DOI:** 10.31744/einstein_journal/2026RW2362

**Published:** 2026-08-21

**Authors:** Yaniv Mayer, Daniela Matanes, Jamil Awad Shibli

**Affiliations:** 1 Technion - Israel Institute of Technology The Ruth and Bruce Rappaport Faculty of Medicine Haifa Israel The Ruth and Bruce Rappaport Faculty of Medicine, Technion - Israel Institute of Technology, Haifa, Israel; 2 Rambam Health Care Campus (RHCC) School of Graduate Dentistry Department of Periodontology Haifa Israel Department of Periodontology, School of Graduate Dentistry, Rambam Health Care Campus (RHCC), Haifa, Israel; 3 Universidade de Guarulhos Dental Research Division Department of Periodontology Guarulhos SP Brazil Department of Periodontology, Dental Research Division, Universidade de Guarulhos, Guarulhos, SP Brazil; 4 Hospital Israelita Albert Einstein Faculdade de Ciências da Saúde Albert Einstein São Paulo SP Brazil Faculdade de Ciências da Saúde Albert Einstein, Hospital Israelita Albert Einstein, São Paulo, SP Brazil; 5 Harvard School of Dental Medicine Department of Oral Medicine, Infection and Immunity Boston Massachusetts United States Department of Oral Medicine, Infection and Immunity, Harvard School of Dental Medicine, Boston, Massachusetts, United States

**Keywords:** Porphyromonas gingivalis, Periodontitis, Cardiovascular disease, Alzheimer's disease, Rheumatoid arthritis, Diabetes mellitus, Adverse pregnancy outcomes

## Abstract

*Porphyromonas gingivalis (P. gingivalis)* is a Gram-negative anaerobic bacterium recognized as a keystone pathogen in periodontitis. Beyond its established role in periodontal tissue destruction, accumulating evidence links *P. gingivalis* to several systemic conditions, including cardiovascular disease, Alzheimer's disease, rheumatoid arthritis, diabetes mellitus, and adverse pregnancy outcomes. This review synthesizes current evidence on how *P. gingivalis* may contribute to systemic disease beyond the oral cavity, with particular emphasis on its virulence factors, including gingipains and lipopolysaccharide, and the pathways linking periodontal infection to pathological processes in distant organs. We discuss how hematogenous dissemination, immune-evasion mechanisms, and chronic inflammatory responses may mediate these systemic effects. A clearer understanding of these associations may inform integrated approaches to periodontal care and systemic disease management.

## INTRODUCTION

### Periodontal disease: definition and pathogenesis

Periodontal diseases comprise a spectrum of inflammatory conditions affecting the supporting structures of the teeth, ranging from gingivitis to severe periodontitis. Periodontitis is among the most prevalent chronic inflammatory conditions in humans, with severe disease estimated to affect approximately 11% of adults worldwide. If untreated, periodontitis can cause progressive periodontal ligament destruction, alveolar bone loss, and ultimately tooth loss.(1)

The pathogenesis of periodontal disease is multifactorial and involves complex interactions among the oral microbiota, host immune responses, and environmental risk factors. Although human subgingival plaque contains more than 500 bacterial species, periodontitis is associated with microbial dysbiosis rather than the simple overgrowth of individual pathogens. This ecological shift involves changes in microbial community structure and an increased abundance of specific pathobionts—organisms that may coexist with the host under homeostatic conditions but promote disease under favorable environmental conditions. (1,2)

### The concept of systemic dissemination

Accumulating evidence of systemic effects has challenged the traditional view of periodontal disease as a strictly localized oral condition.(1,3) In severe periodontitis, the chronically inflamed and ulcerated periodontal pocket may provide a substantial surface area through which bacteria and bacterial products can enter the bloodstream. Routine activities, including toothbrushing, flossing, and mastication, can induce transient bacteremia and permit periodontal bacteria or their products to enter the systemic circulation.

Periodontal bacteria and their products may reach distant tissues through several proposed routes, including hematogenous spread, lymphatic transport, carriage within inflammatory cells, and, in selected experimental contexts, neural pathways. The dense vascular network surrounding periodontal tissues and disruption of the epithelial barrier within inflamed periodontal pockets may facilitate this translocation. Bacterial products, including lipopolysaccharide (LPS), proteolytic enzymes, and outer membrane vesicles (OMVs), may also trigger systemic inflammatory responses associated with increased circulating concentrations of tumor necrosis factor-α (TNF-α), interleukin-6 (IL-6), and C-reactive protein (CRP).(4)

### Porphyromonas gingivalis as a keystone pathogen

#### General characteristics and classification

*Porphyromonas gingivalis* is an obligately anaerobic, asaccharolytic, Gram-negative, nonmotile, rod-shaped bacterium belonging to the phylum Bacteroidota. The organism forms characteristic black-pigmented colonies on blood agar because of its heme-acquisition and storage mechanisms. *P. gingivalis* has a genome of approximately 2.3 million base pairs that encodes 1,990 open reading frames, including an estimated 463 genes considered essential for survival. (5)

Despite its relatively low abundance in the oral microbiome, *P. gingivalis* can exert a disproportionate influence on periodontitis pathogenesis and is therefore regarded as a keystone pathogen.(1) The designation "keystone pathogen" does not imply that *P. gingivalis* alone is sufficient to cause periodontitis. Rather, *P. gingivalis* acts within an established polymicrobial biofilm, where its immune-subversive activities alter microbial community structure and host responses, promoting the transition from symbiosis to dysbiosis and inflammation. Experimental models indicate that *P. gingivalis* does not induce destructive periodontal disease in germ-free hosts or highly simplified microbial communities, underscoring its dependence on other community members for colonization, persistence, and pathogenicity. Thus, the term "keystone pathogen" reflects the organism's disproportionate effects on biofilm ecology and host immunity despite its low relative abundance. At low colonization levels, *P gingivalis* may promote pathobiont expansion and disrupt host-microbe homeostasis throughout the subgingival biofilm. The bacterium exhibits phenotypic plasticity and modifies virulence-factor expression in response to environmental conditions, including heme availability, pH, and nutrient availability.

*P. gingivalis* contributes to periodontitis pathogenesis through several complementary mechanisms. The bacterium subverts host immunity by evading clearance while disrupting protective immune responses. At high heme concentrations, *P. gingivalis* may exert immunosuppressive effects, including downregulation of Toll-like receptor (TLR) signaling, reduced tumor necrosis factor-α (TNF-α) production, and increased interleukin-10 (IL-10) secretion. Conversely, under heme-limiting conditions, the organism may increase its resistance to antimicrobial peptides and enhance gingipain activity, thereby promoting immune evasion.(6)

Its pathogenic mechanisms extend beyond direct tissue destruction. *P. gingivalis* can invade gingival epithelial cells and fibroblasts and persist intracellularly, potentially reducing its exposure to host defenses and antimicrobial agents. Experimental studies indicate that *P. gingivalis* does not induce periodontitis in isolation but depends on commensal bacteria and complement-mediated host responses. These findings support its role as an orchestrator of polymicrobial synergy and dysbiosis rather than as a conventional pathogen acting independently.(5,6)

### Virulence factors

#### Gingipains

Gingipains are among the most extensively studied virulence factors of *P. gingivalis* and contribute substantially to bacterial survival and pathogenicity. These cysteine proteases include the arginine-specific gingipains RgpA and RgpB and the lysine-specific gingipain Kgp; they are secreted through the type IX secretion system (T9SS) and occur in both soluble and membrane-bound forms. Gingipains perform several functions, including degrading host proteins for nutrient acquisition, inactivating host defense molecules, processing bacterial surface proteins, and disrupting epithelial barriers by cleaving junctional proteins such as E-cadherin, β1-integrin, and occludin.(6)

Gingipain proteolytic activity also modulates complement pathways and coagulation cascades. Gingipains can activate platelet protease-activated receptors (PARs), potentially promoting a prothrombotic state. They also degrade immunoglobulins, complement components, and antimicrobial peptides, thereby facilitating immune evasion. Gingipains can also cleave and activate matrix metalloproteinases (MMPs), thereby amplifying tissue damage. The gingipain-associated hemoglobin receptor protein (HbR) domain also functions as an adhesin and can induce interleukin-8 (IL-8) expression in gingival epithelial cells, thereby contributing to local inflammatory responses.(6)

#### Fimbriae

*P. gingivalis* expresses two main types of fimbriae: major fimbriae encoded by fimA and minor fimbriae encoded by mfa1. These surface structures mediate adhesion to host cells, biofilm formation, and immune modulation. FimA interacts with complement receptor 3 (CR3) on macrophages and activates cyclic adenosine monophosphate-dependent protein kinase A (PKA) through CXCR4-TLR2 co-association, thereby inhibiting TLR2-mediated antimicrobial responses. This interaction also promotes ERK1/2 phosphorylation and suppresses interleukin-12 (IL-12), thereby supporting bacterial survival within phagocytes.(5,7)

The minor fimbrial protein Mfa1 likewise activates TLR signaling but may inhibit dendritic-cell autophagy through DC-SIGN-TLR2 crosstalk, thereby facilitating prolonged survival within these antigen-presenting cells. Fimbriae therefore, have dual roles in promoting colonization and modulating host immunity to facilitate persistent infection.(7)

#### Lipopolysaccharide

*P. gingivalis* lipopolysaccharide (LPS) exhibits structural heterogeneity that contributes to its pathogenic effects. Unlike Escherichia coli LPS, *P. gingivalis* LPS varies in lipid A structure and includes both penta-acylated and tetra-acylated forms. This structural diversity alters Toll-like receptor 4 (TLR4) signaling, and some lipid A variants may act as TLR4 antagonists rather than agonists, thereby attenuating protective immune responses. By modulating TLR signaling, *P. gingivalis* may produce a "bystander" effect in which suppression of immune responses to neighboring organisms facilitates expansion of the dysbiotic microbial community.(7)

#### Outer membrane vesicles

Outer membrane vesicles (OMVs) are important vehicles for delivering *P. gingivalis* virulence factors to host tissues. These bilayered membrane structures, which are approximately 50-250nm in diameter, are released from the bacterial surface and contain gingipains, LPS, adhesins, and other virulence-associated molecules. Compared with intact bacteria, OMVs may penetrate otherwise inaccessible tissues, cross physiological barriers such as the blood-brain barrier, evade phagocytic clearance, and deliver molecular cargo to host cells through membrane interactions.(5)

Their potential to disseminate and deliver functional virulence factors to distant sites makes OMVs relevant to the proposed systemic effects of *P. gingivalis.* OMVs can be internalized by several host cell types, including neurons, endothelial cells, and immune cells, where they may induce inflammatory signaling, apoptosis, and other pathological responses.(5)

### Mechanisms of tissue invasion and bloodstream entry

*P. gingivalis* uses several mechanisms to breach epithelial barriers and gain access to the bloodstream. The bacterium invades gingival epithelial cells through a "folding" mechanism that induces marked membrane invagination at the site of entry and leads to internalization within spacious vacuoles. Once internalized, *P. gingivalis* may survive and replicate within epithelial cells, fibroblasts, and endothelial cells, potentially forming intracellular reservoirs that are less accessible to host defenses and antimicrobial agents.(5,6)

Entry into the systemic circulation may occur through several routes (Figure 1). The ulcerated epithelium of periodontal pockets may provide direct access to blood vessels, particularly during mechanically disruptive activities such as chewing, toothbrushing, and dental procedures. The periodontal tissues are also closely associated with lymphatic vessels, which may provide an additional route for bacterial dissemination. In addition, *P. gingivalis* may be transported within inflammatory cells, particularly macrophages and neutrophils, from the oral cavity to distant sites while partially evading immune clearance.(6)

**Figure 1:**
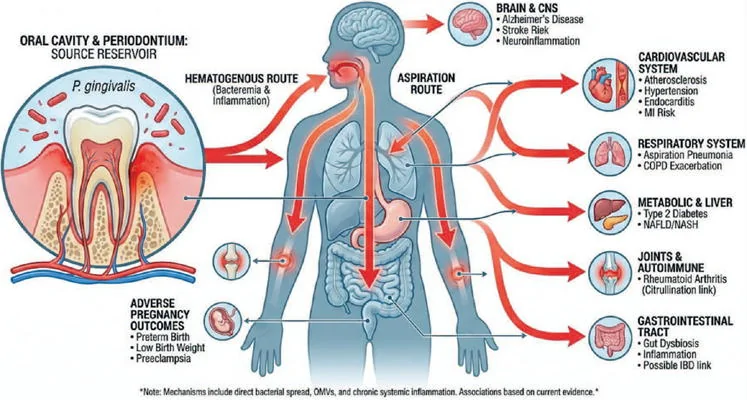
*P gingivalis* originates in the oral cavity and disseminates systemic virulence factors via hematogenous, aspiration, and trans-intestinal routes Primary target organ mechanisms highlighted in the main text include the central nervous, cardiovascular, musculoskeletal, endocrine, and reproductive systems. Note that the figure also depicts respiratory, hepatic, and additional gastrointestinal associations (e.g., aspiration pneumonia, non-alcoholic fatty liver disease, and inflammatory bowel disease) that reflect emerging or preliminary evidence not detailed in the accompanying narrative

Experimental studies also suggest that swallowed *P. gingivalis* can reach the gastrointestinal tract, alter gut microbial composition, and contribute to systemic inflammation through the oral-gut axis. This pathway may represent an additional mechanism through which periodontal infection influences systemic physiology.(8)

### P. gingivalis in systemic diseases: tissue localization and proposed pathological mechanisms

#### Cardiovascular disease and atherosclerosis

Detection in vascular tissues and epidemiological associations *P. gingivalis* DNA, antigens, and, in some studies, viable bacteria have been detected in atherosclerotic plaques, coronary and carotid arteries, and thrombus aspirates from patients with myocardial infarction. Some studies have identified *P. gingivalis* as one of the most frequently detected bacterial species in coronary and femoral arterial specimens from patients with cardiovascular disease. One study detected *P. gingivalis* in arterial specimens from all included patients with cardiovascular disease, although this finding should be interpreted within the context of that study's sample and detection method.(3)

Epidemiological studies have repeatedly identified associations between periodontal disease and increased cardiovascular risk. Patients with periodontitis have shown a higher risk of atherosclerotic cardiovascular disease, myocardial infarction, and stroke in observational studies. Although the American Heart Association recognizes an independent association between periodontal disease and atherosclerotic vascular disease, it has not concluded that the relationship is causal because of shared risk factors and residual confounding(3,9) (Table 2).

**Table 1:** Reported detection of *Porphyromonas gingivalis* in systemic tissues and proposed mechanisms

Disease	Tissue location	Detection method	Detection frequency or epidemiological estimate	Proposed mechanisms
Cardiovascular disease	Atherosclerotic plaques; coronary and carotid arteries	16S rRNA gene sequencing	100% of sampled patients with CVD colonized	Endothelial dysfunction; foam-cell formation; vascular smooth muscle cell calcification; Th17/Treg imbalance
Alzheimer's disease	Hippocampus; cerebral cortex; temporal lobe; cerebrospinal fluid	DNA detection; lipopolysaccharide detection; gingipain immunoreactivity	OR 1.4-1.7 for dementia	Amyloid-β1-42 production; tau cleavage and phosphorylation; blood-brain barrier disruption; neuroinflammation
Rheumatoid arthritis	Synovial fluid; periodontal tissues	Anti-*P. gingivalis* antibodies; PPAD; citrullinated proteins	OR 1.5-2.3 for PD in RA	PPAD-mediated citrullination of fibrinogen, enolase, and collagen; molecular mimicry; ACPA production
Type 2 diabetes	Liver; skeletal muscle; adipose tissue; gastrointestinal tract	Bacterial detection; antibody titers; HOMA-IR	OR 1.5-2.0 for T2D (obese: OR 2.5-3.0);	Bidirectional relationship, Gingipain-mediated insulin receptor degradation; gut dysbiosis; systemic inflammation involving TNF-α and IL-6
Adverse pregnancy outcomes	Placenta; umbilical cord; amniotic fluid	DNA detection by polymerase chain reaction; immunofluorescence	OR 6.73 for preeclampsia; OR 2.1-2.8 PTB 7x PTB risk with low IgG	Localization within the villous mesenchyme; PGE2 and Th1-associated cytokine responses; promotion of preterm labor

Aβ: amyloid-β; ACPA: anti-citrullinated protein antibody; BBB: blood-brain barrier; CSF: cerebrospinal fluid; CVD: cardiovascular disease; HOMA-IR: Homeostatic Model Assessment for Insulin Resistance; IgG: immunoglobulin G; IL: interleukin; INSR nsulin receptor; LPS: lipopolysaccharide; OR: odds ratio; PCR: polymerase chain reaction; PD: periodontal disease; PGE2: prostaglandin E2; PPAD: *P gingivalis* peptidylarginine deiminase; PTB: preterm birth; RA: rheumatoid arthritis; SE: shared epitope. T2D: type 2 diabetes; Th1: T helper 1; TNF-α: tumor necrosis factor-α; VSMC: vascular smooth muscle cell

**Table 2:** Factors potentially modifying associations between periodontal disease and systemic outcomes

Potential modifying factor	Reported association	Effect estimate
Smoking	Cardiovascular disease associated with periodontitis	OR 3.0-1.5 (stratified association)
HLA-DRB1 SE × smoking	ACPA-positive RA (two-way gene-environment interaction)	OR ~7.5
smoking × anti-RgpB	ACPA-positive RA (pairwise interaction	OR ~4.0
Age >65 years	Cardiovascular disease associated with periodontitis	OR 2.0-2.5 vs OR 1.3-1.5 in ≤65 years
APOE ε4 genotype	Alzheimer's disease (or related dementia outcomes) associated with periodontitis	OR 2.5-3.0 (carriers) vs OR 1.4-1.5 (non-carriers)
HLA-DRB1 SE alleles	Periodontal disease prevalence in ACPA-positive rheumatoid arthritis	OR 2.5-3.5
Diabetes (pre-existing) Obesity (BMI >30)	Cardiovascular disease associated with periodontitis obesity as a modifier of the periodontal disease-diabetes association	OR 2.3-2.8 (synergistic effect) OR 2.5-3.0(subgroup estimate)
Low socioeconomic status	Cardiovascular disease associated with periodontal disease preeclampsia associated with periodontal disease	OR 1.5-1.9 OR 2.5-3.0
Duration >10 years	Alzheimer's disease associated with chronic periodontal disease	OR 1.7-2.0 (≥10 years, ICD-9-CM claims-based diagnosis) vs OR 1.2-1.4 (<10 years)

ACPA: anti-citrullinated protein antibody; BMI: body mass index; CVD: cardiovascular disease; OR: odds ratio; PD: periodontal disease; RA: rheumatoid arthritis; SE: shared epitope

#### Proposed pathophysiological mechanisms

*P. gingivalis* may contribute to atherogenesis through several interconnected mechanisms affecting endothelial cells, macrophages, vascular smooth muscle cells, lymphocytes, and platelets:

Endothelial dysfunction: *P. gingivalis* infection can impair endothelial-cell proliferation, promote endothelial-to-mesenchymal transition, and induce apoptosis through activation of the TLR-NF-ΚB axis. The bacterium may disrupt endothelial homeostasis by increasing nitric oxide production through inducible nitric oxide synthase while reducing endothelial nitric oxide synthase activity. Gingipains can increase angiopoietin-2 expression in aortic smooth muscle cells and promote their migration, a process implicated in atherosclerotic plaque development.(10,11)

Foam-cell formation: *P. gingivalis* infection may promote the transformation of macrophages and monocytes into foam cells, a hallmark of early atherosclerotic lesions. The bacterium can increase macrophage uptake of oxidized low-density lipoprotein (oxLDL) by upregulating scavenger receptors. Gingipains may also degrade apolipoprotein A-I, thereby impairing reverse cholesterol transport and promoting lipid accumulation.(12)

Vascular smooth muscle cell alterations: *P. gingivalis* infection may promote the proliferation, migration, and calcification of vascular smooth muscle cells (VSMCs). Under hyperglycemic conditions, the bacterium may enhance VSMC calcification through TLR4 and ERK1/2-p38 signaling, with subsequent activation of bone morphogenetic protein 4 (BMP4) autocrine signaling and the SMAD1/5/8-RUNX2 pathway. In addition, *P. gingivalis* may induce VSMC apoptosis through TLR2 pathway activation, potentially contributing to plaque instability and rupture susceptibility.(10)

Immune-cell dysregulation: *P. gingivalis* infection may disrupt the balance between regulatory T cells (Tregs) and T helper 17 (Th17) cells, thereby promoting a proinflammatory state. The bacterium may reduce Treg abundance and regulatory activity while enhancing Th17 responses and interleukin-17 (IL-17) production. This imbalance may contribute to chronic vascular inflammation and atherosclerotic progression.(10)

Prothrombotic effects: Gingipains can activate platelet protease-activated receptors 1 and 4 (PAR-1 and PAR-4), thereby promoting platelet aggregation. They can also activate coagulation factors II, IX, and X through proteolytic cleavage, thereby creating a prothrombotic milieu that may increase susceptibility to thrombotic complications, including myocardial infarction and stroke.(6)

Animal models provide mechanistic evidence that supports a potential causal contribution. In pigs, repeated intravenous administration of *P. gingivalis* induced coronary and aortic atherosclerotic changes in both normocholesterolemic and hypercholesterolemic animals. Similarly, oral infection with *P. gingivalis* accelerated atherosclerosis in apolipoprotein E-deficient mice and was associated with mortality from cardiac rupture in experimental models.(13)

#### Alzheimer's disease and neurodegeneration

Detection in brain tissue and cerebrospinal fluid Studies have detected *P. gingivalis* DNA, lipopolysaccharide (LPS), and gingipains in postmortem brain tissue from patients with Alzheimer's disease (AD). Gingipains have been detected in several brain regions, including memory-associated areas such as the hippocampus. Gingipain immunoreactivity has been reported to correlate with AD diagnosis and the severity of tau and ubiquitin pathology. Specifically, RgpB and Kgp have been detected in association with neurons, tau tangles, and amyloid-β plaques, findings that support a possible role in AD-related pathological processes.(14,15)

*P. gingivalis* DNA has also been detected in the cerebrospinal fluid of patients with AD, although this finding alone does not establish viable or active brain colonization. *P. gingivalis* has been detected more frequently in brain tissue from patients with AD than in age-matched controls without cognitive impairment, although lower-frequency detection in some control brains suggests that bacterial burden, tissue distribution, or host susceptibility may influence its pathological relevance.(14)

#### Proposed mechanisms of brain entry and neurodegeneration

*P. gingivalis* and its virulence factors may reach the brain through several proposed pathways. Disruption of the blood-brain barrier (BBB) is one proposed mechanism, as gingipains can cleave barrier-associated junctional proteins, including E-cadherin, β1-integrin, and occludin. BBB dysfunction becomes more pronounced with aging and in carriers of the APOE ε4 allele, a major genetic risk factor for AD, and may partly explain stronger associations between periodontitis and dementia in susceptible populations.(14,16)

Outer membrane vesicles (OMVs) may serve as vehicles for delivering bacterial components to the brain. Experimental evidence suggests that these vesicles can cross the BBB and deliver gingipains and LPS to brain tissue. In addition, *P. gingivalis* may access the brain through trigeminal pathways, as experimental oral infection can involve primary afferent nerves that project to regions including the locus coeruleus and hippocampus(14) (Figure 2).

**Figure 2:**
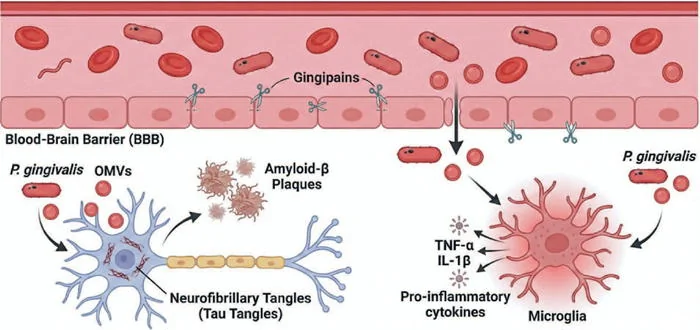
Proposed involvement of *Porphyromonas gingivalis* in Alzheimer's disease pathology

Within the brain, *P. gingivalis* and its virulence factors may contribute to several neurodegenerative processes:

Amyloid-β accumulation: In mice, oral *P. gingivalis* infection has been associated with bacterial detection in the brain and increased production of Aβ1-42, a highly amyloidogenic form of amyloid-β. Chronic exposure to *P. gingivalis* LPS can promote amyloid-β accumulation in brain tissue and peripheral inflammatory monocytes and macrophages, suggesting that these cells may act as circulating carriers or reservoirs. Because amyloid-β may have antimicrobial activity, its accumulation has been proposed as a host response to chronic infection.(17)

Tau pathology: Gingipains can cleave tau protein and generate fragments containing the VQIINK and VQIVYK hexapeptide motifs found in paired helical filaments within neurofibrillary tangles (NFTs). Beyond direct proteolysis, *P. gingivalis* infection may promote tau phosphorylation by activating inflammatory pathways involving glycogen synthase kinase-3β (GSK-3β). Hyperphosphorylated tau has reduced microtubule-stabilizing capacity, contributing to neuronal dysfunction and aggregate formation.(14)

Neuroinflammation: *P. gingivalis* OMVs and LPS can activate microglia and astrocytes and induce production of proinflammatory cytokines, including TNF-α, IL-1β, and IL-6. Sustained neuroinflammation may contribute to synaptic dysfunction, neuronal injury, and cognitive decline. The bacterium may also activate the NLRP3 inflammasome in brain-resident immune cells, thereby amplifying inflammatory responses.(14,18)

Iron dysregulation and ferroptosis: *P. gingivalis* has extensive iron-acquisition systems, and its presence in brain tissue may disrupt iron homeostasis. It has been hypothesized that iron-rich deposits in the AD brain may create an environment that favors bacterial persistence or virulence. Dysregulated iron metabolism can induce ferroptosis, an iron-dependent form of regulated cell death that may contribute to neuronal loss.(14)

Animal studies provide mechanistic evidence supporting a potential causal contribution. Oral infection of wild-type and ApoE-deficient mice with *P. gingivalis* has been associated with bacterial detection in the brain, neuroinflammation, amyloid deposition, microglial activation, tau-related pathology, and neurodegeneration. In experimental models, gingipain inhibitors have reduced brain bacterial burden, amyloid-β production, neuroinflammation, and hippocampal neuronal loss, supporting further investigation of this therapeutic strategy.(17,19)

Oral *P. gingivalis* and its outer membrane vesicles (OMVs) may enter the systemic circulation, cross or disrupt the blood-brain barrier (BBB), and activate cerebral innate immune responses. Microglial activation, pro-inflammatory cytokine release, and systemic neurovascular interactions may promote neuroinflammation, neurotoxicity, amyloid-β and tau pathology, synaptic dysfunction, and neuronal loss, thereby contributing to cognitive decline and disease progression. Teal elements indicate findings supported by human tissue or clinical studies, lavender elements denote mechanisms derived mainly from animal or *in vitro* evidence, and dashed arrows represent proposed feedback or bidirectional interactions.

### Rheumatoid arthritis

#### Epidemiological associations

Rheumatoid arthritis (RA) is a chronic autoimmune disease characterized by synovial inflammation, progressive joint destruction, and autoantibodies against citrullinated proteins (ACPAs). Epidemiological studies have repeatedly reported a higher prevalence of periodontal disease among patients with RA than among controls and, conversely, a higher prevalence of RA among patients with periodontitis. Patients with RA show antibodies against *P. gingivalis* more frequently than controls, and elevated antibody levels may precede the clinical onset of RA by several years.(20)

#### The PPAD-citrullination hypothesis

*P. gingivalis* has been described as the only known prokaryote that expresses a peptidylarginine deiminase, termed *P. gingivalis* peptidylarginine deiminase (PPAD). This bacterial enzyme catalyzes protein citrullination, the post-translational conversion of arginine residues to citrulline. Unlike human PADs, which commonly target internal arginine residues, PPAD preferentially modifies C-terminal arginine residues generated by gingipain-mediated cleavage.(21)

The PPAD-citrullination hypothesis proposes that *P. gingivalis* may disrupt immune tolerance to citrullinated proteins in susceptible individuals, thereby promoting ACPA production and contributing to RA development. Established RA-related autoantigens that can undergo citrullination include fibrinogen, a-enolase, vimentin, and type II collagen. Surface-associated PPAD may citrullinate host proteins that interact with *P. gingivalis* through bacterial adhesins. *P. gingivalis* can citrullinate fibrinogen-derived peptides and α-enolase, both of which are established RA autoantigens.(22,23)

The citrullinated enolase peptide-1 (CEP-1) epitope shows 82% sequence similarity between human and *P. gingivalis* α-enolase, thereby providing a potential basis for molecular mimicry. Antibodies against CEP-1 are associated with RA and may cross-react with *P. gingivalis*-derived enolase. Elevated antibody responses to citrullinated PPAD peptides have been detected in serum from patients with RA, particularly against the CPP3 peptide, which showed reactivity in approximately 40% of samples in one study.(24)

Animal models support a potential causal contribution of *P. gingivalis* to arthritis progression. Infection with viable *P. gingivalis* exacerbates collagen-induced arthritis (CIA) in mice, resulting in earlier onset, more rapid progression, greater disease severity, and increased bone and cartilage destruction. This arthritogenic effect appears to depend on PPAD expression, as infection with PPAD-null mutants does not exacerbate arthritis in these models. *P. gingivalis* infection increases autoantibodies against type II collagen and citrullinated epitopes, while high concentrations of citrullinated proteins are detected at periodontal infection sites.(22)

Figure 3 summarizes the proposed molecular mechanism linking *P. gingivalis* to rheumatoid arthritis (RA). The diagram shows PPAD converting arginine residues in host proteins to citrulline. This process generates citrullinated proteins or neoepitopes that may disrupt immune tolerance and promote ACPA production by B cells and plasma cells. The resulting ACPAs may bind to citrullinated proteins within the joint and contribute to inflammation and tissue destruction characteristic of RA (Figure 3).

**Figure 3:**
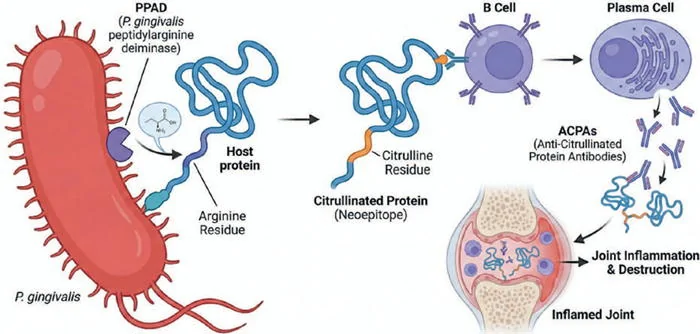
Proposed PPAD-citrullination pathway in rheumatoid arthritis

#### Additional proposed mechanisms

Beyond PPAD-mediated citrullination, *P. gingivalis* may contribute to RA through chronic inflammation and immune dysregulation. Periodontitis-associated inflammatory mediators, including TNF-α and IL-17, can exacerbate experimental arthritis. In mice with pre-existing periodontitis, *P. gingivalis* infection increases TNF-α and IL-17 concentrations and is associated with more severe joint damage. This effect is attenuated by IL-17 receptor A deficiency, suggesting that periodontitis-related cytokine signaling contributes to arthritis exacerbation. Clinical studies have reported that anti-TNF-α therapy improves joint disease activity and may also improve periodontal parameters in patients with RA and other autoimmune diseases.(25,26) In addition, *P. gingivalis*-induced NETosis, or neutrophil extracellular trap formation, may provide another source of citrullinated antigens and inflammatory mediators relevant to RA pathogenesis.(20)

### Diabetes mellitus and metabolic disorders

#### Bidirectional relationship between periodontitis and diabetes

The relationship between periodontal disease and diabetes is bidirectional: diabetes increases susceptibility to periodontitis, whereas periodontitis may adversely affect glycemic control. *P. gingivalis* has emerged as a potential mediator of this relationship, with accumulating evidence suggesting a contribution to insulin resistance and impaired glucose metabolism.(27)

#### Proposed mechanisms of insulin resistance

*P. gingivalis* may contribute to insulin resistance through several interconnected mechanisms. Direct insulin-receptor degradation: Experimental studies indicate that *P. gingivalis* can reach insulin-responsive tissues, including the liver, skeletal muscle, and adipose tissue. Gingipains can directly degrade the insulin receptor (INSR) through proteolytic cleavage of its functional insulin-binding region. This mechanism provides a possible molecular explanation for how distant *P. gingivalis* exposure may impair systemic insulin signaling. Clinical data also show a correlation between *P. gingivalis* abundance in periodontal pockets and Homeostatic Model Assessment for Insulin Resistance (HOMA-IR) scores.(28) Similarly, periodontal bone loss has been associated with impaired fasting glucose and unfavorable serum lipid profiles even in adults without diabetes, suggesting that periodontal disease may accompany early metabolic dysregulation before overt diabetes develops.(29)

Systemic inflammation: *P. gingivalis* infection may increase circulating proinflammatory cytokines, including TNF-α, IL-6, and IL-17, which can interfere with insulin signaling. In adipose tissue, exposure to *P. gingivalis* LPS can increase secretion of resistin and leptin, which are associated with insulin resistance, while reducing adiponectin, an insulin-sensitizing adipokine. The bacterium may also upregulate inflammatory mediators, including angiopoietin-like 4 (ANGPTL4), which has been implicated in insulin resistance.(27)

Gut microbiome dysbiosis: In animal models, oral administration of *P. gingivalis* alters gut microbial composition, including the relative abundance of taxa within the phylum Bacteroidota and genera such as Prevotella. These microbial changes are associated with increased systemic inflammation and insulin resistance. *P. gingivalis* may induce enterohepatic metabolic disturbances by altering intestinal metabolites and hepatic gluconeogenic pathways. The bacterium has also been detected in fecal specimens after oral administration, supporting gastrointestinal passage or translocation.(8,30,31)

Metabolic syndrome: In mice fed a high-fat diet, *P. gingivalis* infection induces features associated with metabolic syndrome, including increased fat mass, adipose-tissue inflammation, elevated fasting glucose, insulin resistance, and glucose intolerance. Patients with metabolic syndrome have shown elevated antibody titers against *P. gingivalis,* and these titers correlate positively with markers of insulin resistance. Toothbrushing frequency has been inversely associated with the incidence of metabolic syndrome, although this observational relationship does not establish causality.(30)

#### Impact on glucose metabolism

Animal studies indicate that oral administration of *P. gingivalis* can impair glucose metabolism. Infected mice develop impaired glucose tolerance and reduced insulin sensitivity, accompanied by altered expression of glucose metabolism-related genes in the liver and adipose tissue. The bacterium can increase the expression of gluconeogenesis-related enzymes and alter associated metabolites while enhancing Th17 responses and inflammatory chemokines, including CCL2, CCL8, and CXCL10. In experimental models, gingipain-deficient *P. gingivalis* produces less pronounced metabolic impairment, supporting a mechanistic role for these virulence factors.(32)

#### Adverse pregnancy outcomes

Adverse pregnancy outcomes associated with P. gingivalis *P. gingivalis* has been associated with several adverse pregnancy outcomes (APOs), including preterm birth, low birth weight, preeclampsia, fetal growth restriction, and spontaneous abortion. *P. gingivalis* DNA and antigens have been detected in placental tissue, amniotic fluid, umbilical-cord specimens, and neonatal nasogastric aspirates from complicated pregnancies, with some studies reporting higher detection frequencies or microbial burdens than in uncomplicated pregnancies.(33,34,35)

#### Proposed mechanisms and pathways

*P. gingivalis* may reach the fetoplacental unit through hematogenous dissemination from periodontal tissues. Once present, the bacterium may directly affect uteroplacental tissues or indirectly contribute to adverse outcomes through systemic inflammatory mediators.(33)

Preeclampsia: Detection of *P. gingivalis* in umbilical-cord tissue has been associated with preeclampsia (odds ratio [OR], 6.73; 95% confidence interval [95%CI], 1.31-36.67) though the wide confidence interval reflects the limited sample size and warrants cautious interpretation. Localization within the placental villous mesenchyme, rather than only at the syncytiotrophoblast surface, may be particularly relevant to adverse outcomes. *P. gingivalis* may contribute to preeclampsia through endothelial dysfunction, systemic inflammation, and impaired placental angiogenesis.(33)

Preterm birth: *P. gingivalis* infection may enhance T helper 1-associated cytokine responses, including interferon-γ, IL-2, IL-12, and TNF-α, and increase prostaglandin E2 (PGE2) concentrations. These inflammatory mediators may promote cervical ripening and uterine contractions, thereby contributing to preterm labor. Women with preterm birth have shown higher gingival crevicular-fluid concentrations of IL-1β, IL-6, and PGE2 than comparison groups. Women with low serum anti*-P. gingivalis* immunoglobulin G titers had an approximately sevenfold higher odds of preterm birth in a case-control study of 124 pregnant/ postpartum women, suggesting that stronger antibody responses may be protective.(35)

Fetal growth restriction: *P. gingivalis* infection may contribute to this outcome through placental dysfunction, impaired angiogenesis, and chronic maternal inflammation. Animal models indicate that maternal *P. gingivalis* infection can result in fetal growth restriction.(34)

Table 1 summarizes reported detection of *Porphyromonas gingivalis* in systemic tissues and the associated proposed mechanisms.

## LIMITATIONS AND UNCERTAINTY

Whether *P. gingivalis* has a causal role in adverse pregnancy outcomes remains uncertain. Periodontal disease alone is a poor predictor of individual pregnancy complications, and randomized trials of periodontal treatment during pregnancy have not consistently reduced preterm birth or other adverse outcomes. Possible explanations include treatment being initiated after relevant inflammatory or immune changes have occurred and incomplete elimination of periodontal bacterial reservoirs. In addition, *P. gingivalis* has been detected by immunofluorescent histology in some placental specimens from uncomplicated pregnancies, although generally at lower frequencies and detection alone does not confirm viable organisms. localization within deeper villous tissue rather than only the surface may reduce, but does not eliminate, the possibility of specimen contamination. These findings suggest that microbial load, tissue localization, or methodological factors may influence the observed association.(33,35,36)

## CONCLUSIONS AND CLINICAL IMPLICATIONS

Accumulating evidence indicates that *P. gingivalis* may influence pathological processes beyond periodontal tissues. Through immune-evasion mechanisms, multiple virulence factors, and environmental adaptability, this keystone pathogen may promote local dysbiosis and gain systemic access to distant organs and tissues. *P. gingivalis* or its components have been detected in atherosclerotic plaques, brain tissue, synovial fluid, pancreatic tissue, and placental specimens, with mechanistic evidence of varying strength supporting possible pathological effects at these sites.(1,4)

Proposed mechanisms linking *P. gingivalis* to systemic disease include direct bacterial effects, such as tissue invasion, protease activity, and receptor degradation; indirect inflammatory effects, including cytokine release, immune dysregulation, and complement activation; and alterations in host physiology, including endothelial dysfunction, insulin resistance, and protein citrullination. The relative contribution of each mechanism likely varies according to the disease context and individual susceptibility factors, including genetic background, immune status, and environmental exposures.(1,6) Although this review focuses on *P gingivalis,* its growth, virulence-factor expression, and systemic dissemination should be understood within the context of polymicrobial synergy and dysbiosis rather than as the effects of an isolated single-species infection. Thus, *P. gingivalis* is more appropriately considered a keystone orchestrator of a dysbiotic community than an independent causal agent.

From a clinical perspective, these findings support periodontal health maintenance as a component of comprehensive care, although its effectiveness in preventing systemic disease remains unconfirmed.

These considerations align with current periodontitis-management principles, which emphasize comprehensive diagnosis, risk-factor control, and staged nonsurgical and surgical therapy to limit local tissue destruction and potentially reduce systemic inflammatory burden.(37) Patients with cardiovascular disease, diabetes, RA, cognitive impairment, or high-risk pregnancies may benefit from periodontal assessment and appropriate treatment as part of coordinated multidisciplinary care, though current obstetric guidance does not support periodontal treatment specifically to reduce adverse pregnancy outcomes. Conversely, patients with severe or treatment-resistant periodontitis may warrant evaluation for relevant systemic comorbidities that could influence periodontal inflammation or treatment response.(38,39)

Therapies targeting *P. gingivalis* virulence factors, particularly gingipains, represent a potential avenue for future periodontal and systemic-disease research. Preclinical studies of gingipain inhibitors have shown reductions in bacterial burden and neuroinflammation, but clinical efficacy remains unestablished.(14)

Future research should move beyond cross-sectional and associative evidence toward longitudinal studies, mechanistically informed interventions, and risk-stratified approaches. Potential strategies include small-molecule gingipain inhibitors and vaccines targeting virulence factors such as PPAD and fimbriae, with the aim of limiting pathogenic effects while preserving the commensal oral microbiome. Large longitudinal cohorts are also needed to clarify temporal relationships and strengthen causal inference between periodontal infection and systemic disease onset. Validation of biomarkers, including specific antibody titers and salivary pathogen burden, may also support future risk-stratification strategies. These efforts may support integrated healthcare models in which periodontal assessment and maintenance complement established strategies for cardiovascular, metabolic, and neurodegenerative disease management.

A clearer understanding of the interactions among *P. gingivalis,* host immunity, and systemic disease may inform more effective prevention and treatment of periodontal disease and its associated systemic conditions.

## Data Availability

The underlying content is contained within the manuscript.
